# Pituitary cells in man during aging: An immunohistological and morphometric study

**DOI:** 10.5937/jomb0-54605

**Published:** 2025-03-21

**Authors:** Jovana Čukuranović-Kokoris, Braca Kundalić, Miljana Pavlović, Sladjana Ugrenović

**Affiliations:** 1 University of Niš, Faculty of Medicine, Department of Anatomy, Niš

**Keywords:** ACTH cells, GH cells, LH cells, FS cells, aging, ACTH ćelije, GH ćelije, LH ćelije, FS ćelije, starenje

## Abstract

**Background:**

Aging is a multifactorial process, which inevitably leads to numerous changes in the life of each individual. As the lifespan of people around the world has increased, scientists began to study the mechanisms that would enable high-quality and safe aging. In this review, the focus is on showing the effect of aging on the characteristics of pituitary cells.

**Methods:**

In order to write this review, we undertook an electronic literature search through the PubMed and Google Scholar databases using the terms aging, histological, ultrastructure of pituitary cells, also using the "related articles" option. We also analyzed authored and co-authored articles, which were necessary for this type of publication.

**Results:**

This review article describes the immuno-histological, ultrastructural and morphometric characteristics of adrenocorticotropic (ACTH), somatotropic (GH), gonadotropic (LH) and folliculostellate (FS) pituitary cells during aging, in young (about 47 years) and old (over 70 years) human cadavers.

**Conclusions:**

The described immunohistological and morphometric characteristics of pituitary cells in this review showed that the volume density of ACTH, GH and LH cells was statistically increased (p<0.05) in old cadavers, which indicates an attempt by these cells to bring the pituitary gland to a relatively balanced state.

## Abstract (sr)

**Uvod:**

Starenje je multifaktorski proces, koji neminovno dovodi do brojnih promena u životu svakog pojedinca. Kako se životni vek ljudi širom sveta produžio, naučnici su počeli da proučavaju mehanizme, koji bi omogućili kvalitetno i bezbedno starenje. Cilj pregleda je da ukaže na promene u ćelijama hipofize izazvane procesom starenja.

**Metode:**

Za pisanje ovog pregleda korišćena je elektronska pretraga literature kroz baze podataka PubMed i Google Scholar sa terminima starenje, histološki, ultrastruktura ćelija hipofize, a korišćena je i opcija "srodni članci", kao i dodatna analiza autora i koautora članaka koji su neophodni za ovu vrstu publikacije.

**Rezultati:**

U ovom preglednom članku opisan je uticaj starenja na imunohistološke, ultrastrukturne i morfo-metrijske karakteristike folikulostelatnih (FS), adreno kortikotropnih (ACTH), somatotropnih (GH) i gonadotropnih (LH) celija hipofize, kod mladih (oko 47 godina) i starih (preko 70 godina) ljudskih leševa.

**Zaključak:**

Opisane imunohistološke i morfometrijske karakteristike ćelija hipofize u ovoj reviji, ukazuju na značajno povećanje zapreminske gustine ACTH, GH i LH ćelija kod starih kadavera, što je pokazatelj pokušaja ovih ćelija da dovedu hipofizu u kakvu-takvu ravnotežu.

## Introduction

The pituitary gland represents a structural and functional link between the two main regulatory integration systems of the human body - nervous and endocrine. The pituitary gland is an endocrine gland that controls almost the entire endocrine system and affects the growth, metabolism, stress response, and reproduction of the organism [1]. The glandular part is represented by the adenohypophysis, while its nervous part is the neurohypophysis [2]. Adenohypophysis makes up about 75% of the entire gland and consists of the distal, intermediate, and tuberal parts. The intermedial and tuberal parts are stunted in humans, so endocrine activity is almost exclusively associated with the *pars distalis* of the adenohypophysis [3]. Cells that produce hormones that secrete adrenocorticotropic hormone (ACTH), thyroid stimulating hormone (TSH), gonadotropic hormones (follicle stimulating hormone-FSH and luteinizing hormone-LH), growth hormone (GH), prolactin (PRL) and human chorionic gonadotropin (hCG) are located in the distal part of the pituitary gland [1]. The pars intermedia in humans is very poorly developed and makes up only 2% of the pituitary gland. It is made up of cells that secrete melano-stimulating hormone (MSH) and folliculostellate cells [4]. MSH cells are large and polygonal [5]. The granular endoplasmic reticulum (gER) is poorly developed and consists of individual, rare, narrow cisterns. Cisternae, vesicles and vacuoles are differentiated in the Golgi zone, and few mito chondria are elongated [6]. The *pars tuberalis* is a small area around the infundibulum of the pituitary gland. Epithelial cells in this sheath are folliculostellate cells [4]. Alpha-Melanocyte-stimulating hormone (α-MSH) is involved in melanogenesis [7].

Aging is a multidimensional process that involves changes in the interaction of social, psychological, cognitive and genetic aspects [8]. Many works point to the fact that with aging, there are structural changes in ACTH cells, gonadotropic LH cells and somatotropic cells of the adenohypophysis of humans and animals, which can lead to the aforementioned hormonal dysfunction [9] [10] [11] [12] [13] [14] [15]. In different people, aging processes are significantlydifferent, and they are conditioned by life habits and environmental factors [16]. Physiological changes in the functioning of the hypothalamus and pituitary gland (HP) are responsible for the mechanism of biological aging [10]. A large number of studies indicate that with aging, the hypothalamic–pituitary–adrenal axis (HPA) system becomes more sensitive to stressors and that its return to normal functioning is slower [17]. In addition to the HPA axis, significant changes occur in the functioning of the gonadotropic and somatotropic axes [18] [19]. The term gonado-pause [18] generally describes a decrease in the bioavailability of sex hormones, while the term somatopause [19] describes a progressive decline in the bioavailability of GH and/or insulin-like growth factor 1 (IGF-1).

Although the role of the pituitary gland is essential for the balanced functioning of the organism, there are few studies dealing with the structural changes of pituitary cells during aging [7].

### Ontogeny of ACTH, GH, FSH, LH, and FS cells during aging

ACTH cells were immunocytochemically detected within Rathke’s diverticulum, while the concentration of ACTH was determined by radioimmunoassay in fetal blood up to the eighth week of gestation. ACTH-positive cells appear after 21 weeks of gestation in the middle part of the human fetal pituitary gland [20]. In the anterior pituitary of adults, ACTH makes up between 10% and 20% of the cell population and can be single or in groups [10] [21]. These cells synthesize the precursor molecule ACTH known as proopiomelanocortin (POMC), from which the following are produced by the action of proteolytic enzymes within corticotrophs: β-lipotropic hormone (LPH), β-endorphin, ACTH, enkephalin and melanocyte-stimulating hormone (MSH) [10].

Secretion of pituitary *GH cells* increases during gestation and decreases in the neonatal period dueto the activation of the negative feedback mechanism by increasing concentrations of IGF-1 [22]. GH secretion remains stable during childhood, increases during puberty, and then decreases during adulthood [23]. The level of circulating GH after 30 years of age decreases by 1% per year so that in older men, the release of GH can be 5 to 20 times lower than that in younger adults [24], indicating that GH production decreases by 14% every ten years; therefore, the deficiency of this hormone can be over 35% in men aged 60 years. The level of GH in people older than 70 years is significantly reduced and is approximately 1/3 of the recorded values at puberty [25]. Thecauses and mechanisms responsible for the development of late hyposomatotropism, i.e. somatopause, have not been sufficiently studied and described in the literature. Decreased GH secretion during aging is thought to result from a combination of decreased secretion of GH-releasing hormone (GHRH) or ghrelin [26] as well as excessive secretion of somatostatin (SRIH) [27]. All these factors lead to a reduced amount of secreted GH per secretory impulse. Aging does not change the frequency of pulsatile GH secretion, nor does it affect the basal secretion of GH and its half-life, nor the kinetics of its elimination [28]. Changes in GH secretion with aging are partly due to specific structural changes at the level of the hypothalamus [29] but also to some insufficiently studied factors that affect the somatotropes of the adenohypophysis [30]. The consequences of somatopause are reflected in the appearance of intra-abdominal obesity, osteoporosis, insulinresistance, hyperlipidemia, risk of atherosclerosis and cardiovascular diseases [31]. Since the clinical signs of late hypogonadism and hyposomatotropism significantly overlap, some authors believe that they are the result of a common pathophysiology, i.e. that the decrease in GH/IGF-1 secretion is the result of decreased androgen secretion that decreases with age and decreases hypothalamic-pituitary GH function/IGF-1 axis [32].

Gonadotropic cells (FSH and LH) in humans make up 7–15% of the distal part of the pituitary gland [33]. In the fetal hypothalamus, gonadotropic releasing hormone (GnRH) is present from 6 weeks of gestation [34]. Measurable amounts of LH and FSH in the fetal pituitary are up to 10 weeks, and the first circulating gonadotropins in the human fetus have been observed up to 12–14 weeks of gestation [35]. In the serum, the highest levels of FSH and LH are detected around 20 weeks [36]. The con centration of these hormones decreases in the second half of pregnancy, triggering negative feedback mechanisms, increasing maternal estrogen, as well as the secretion of sex steroids by the fetal gonads [34]. Postnatal serum LH and FSH levels peak at around 2-3 months of age and then return to the prepubertal level at about 6 months of age in boys and 1–2 years of age in girls [37]. During childhood, gonadotropin concentrations do not change until the onset of puberty, while serum LH and FSH levels increase during puberty [38]. Due to changes in the functioning of the hypothalamus-pituitary-gonadal axis a decrease in testosterone levels was found in older men both due to testicular anomalies and higher basal levels of LH and FSH [39]. Serumtestosterone concentration in men older than 70 years progressively decreases by almost 25% [40]. Due to disturbances in the functioning of the hypothalamus-pituitary-gonadal axis, due to reduced secretion of gonadotropin-releasing hormone (GnRH), occurs gonadopause (late hypogonadism) [12] [41]. Late hypogonadism is characterized by abdominal obesity, reduced beard growth, bone and muscle mass [42]. In addition to the mentioned typical characteristics of hypogonadism, there are also numerous non-specific symptoms such as depression, memory loss, nervousness, insomnia, temporary hot flashes or sweating, as well as a loss of sexual desire [18]. The daily rhythm of testosterone secretion, observed in younger men, may be reduced or lost in older men [40]. The bioavailability of free testosterone in relation to total testosterone decreases with age because the level of globulins that bind sex hormones decreases [39]. Decreased serum testosterone concentrations due to age are mainly due to reduced production, as its plasma clearance rates are lower in older men than in younger men due to the combined effects of medications, comorbid conditions, and intercurrent diseases [43]. In older men, established lower testosterone concentrations are mostly associated with preserved, and sometimes increased, but irregular LH secretion [44]. The causes of dysregulated LH secretion in older men and testosterone release are still unclear. One reason may be impaired testosterone feedback efficiency due to reduced expression of androgen receptors in the brain and pituitary gland [27]. Synthesis and secretion of LH are, in addition to central regulation, also under the control of various signalling molecules involved in paracrine/autocrine control of gonadotropic function [45]. Gonadotropes interact with the hormone-producing GH, ACTH, and PRL cells of the pituitary gland [11], as well as with non-hormonal cells such as folliculostellate cells [17].

### Immunohistological characteristic of ACTH, GH, LH, and FS cells in cadavers during aging

**Figure 1:**
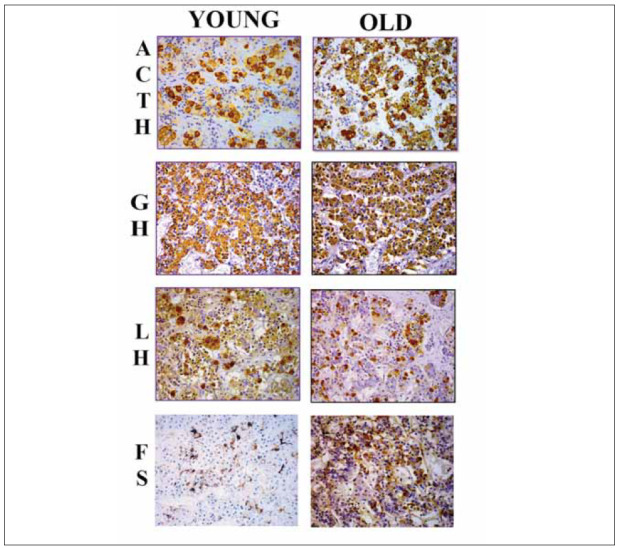
Representative micrography of adrenocorticotropic (ACTH), somatotropic (GH), gonadotropic (LH) and folliculostellate (FS) cells in the pars distalis of the adenohypophysis in a young (44-year old) and an old (80-year old) man; Peroxidase–antiperoxidase (PAP) technique 40 × lens magnification.

*Immunopositive ACTH* cells are oval, polygonal or star-shaped, brown, with a pronounced cytoplasm. The nuclei are euchromatic, eccentrically located, spherical or irregular. These cells are mainly located in the central, mid-sagittal region, less so in the wings of the distal part of the pituitary gland and only occasionally in the tuberal region. In the glandular acinus of young men, ACTH cells are negatively stained with small clusters of positively labelled single cells in between (Figure 1) [10]. Immunopositive ACTH cells of the middle wedge of the distal pituitary gland in older cases are more numerous than in young cases. ACTH cells are distributed in oval structures or larger irregular groups [10]. Ultrastructural analysis showed that secretory granules with a diameter of 200–350 nm are distributed along the cell membrane. The granular endoplasmic reticulum (gER) is poorly developed in contrast to the well-developed Golgi complex. A few mitochondria are rod-shaped, with transverse cristae [6].

*Somatotropic cells* of younger corpses are more prominent in the lateral wings of the adenohypophysis than in the intermedial part. They are predominantly polygonal in shape with an eccentric euchromatic nucleus, and immunopositivity is observed in the cytoplasm [14] (Figure 1). In older cases, there are significantly fewer somatotropes in the intermediate part and lateral wings of the adenohypophysis. The cells showed a slightly stronger immunopositive response in the pars intermedia of the adenohypophysis [10]. Earlier immuno-electronmicroscopic studies of the rat pituitary showed that GH cells are pyramidal, usually in groups, in close contact with blood capillaries [46]. According to the size of the secretory granule, rat GH cells are divided into three types. Type I GH cells contain large secretory granules (diameter 250–350 nm) and are numerous after birth and up to 6 months of age. Type II GH cells contain small (diameter 100–150 nm) and large secretory granules and are present after 6 months of age. Type III cells are characterized by small secretory granules and are immature GH type cells [47]. The granular endoplasmic reticulum is well developed in GH cells. The Golgi complex is located near the nucleus and is moderately developed, near which there are vesicular and vascular structures. Elongated mitochondria are present in moderate numbers and are distributed around the nucleus [48].

In younger cadavers (41 years old), immuno-positive *gonadotropic LH cells* were observed in thelateral wings of the adenohypophysis [49], oval or polygonal in shape and often with an irregularly positioned, euchromatic nucleus, immunopositive cytoplasm, brown in color, with numerous secretory granules (Figure 1) [6]. In older cases, a reduced presence of blood vessels and interstitial fibrosis is observed in the pituitary gland, and the distribution and presence of LH cells are similar to that of younger cases [12]. Oval cells were larger than in younger cases, with an immunonegative eccentrically placed nucleus. The immunopositivity of old and young cadavers was similar (Figure 1) [18] [49]. On electron microscopy, an eccentrically placed spherical nucleus can be seen, the Golgi zone is very dispersed, mitochondria are rare with lamellar cristae, poorly developed rough endoplasmic reticulum (RER) is detected [50]. Numerous secretory granules are basophilic and can be smaller (diameter 200–250 nm), which are rare and larger, with a diameter of about 350–500 nm. These cells are among the largest cells in the pituitary gland [32].

Folliculostellate cells are undifferentiated stem cells that, under specific conditions, can differentiate into different forms of pituitary cells [12]. Immunohistologically labelled folliculostellate cells of the anterior pituitary gland in younger cadavers are stars-haped, with processes that stretch between endocrine cells. In the lateral wings, as well as in the mucoid wedge, the cells were rare and irregularly distributed in the lateral wings. The bodies of FS cells or their extensions are observed in the mucoid wedge, together with endocrine cells, inside the walls of the follicular formation. In the group of oldest cases, it was noticed that FS cells were irregular inshape, with a significant increase in their number both in the middle and in the lateral wings of the adenohypophysis, in addition to a further increase in their size compared to younger cases [14]. Folliculostellate cells form a mesh structure that pervades the entire pituitary gland. In humans, these cells form a wall around pseudofollicular cells – they are small in volume, with scant cytoplasm. The ultra structure of these cells is microfilamentous [4]. Rare lipid droplets can be found in the cytoplasm of these cells. Golgi complex, gER are poorly developed. Mitochondria are small, rare and non-specific structures [4].

### Morphometric characteristic of ACTH, GH, LH, and FS cells during aging

**Figure 2:**
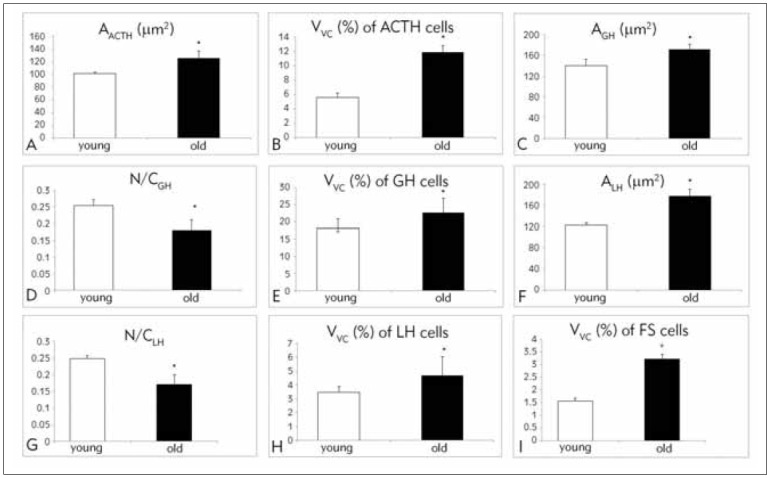
Graphical representation of the anterior pituitary gland parameters in the analyzed cases: correlation between age and area (A, C, F), volume density (B, E, H, I), nuclear-cytoplasmic ratio (D, G); A_ACTH_ – area of corticotropic cells; V_VACTH_ – volume density of corticotropic cells; A_GH_ – area of somatotropic cells; (N/C)_GH_ – nuclear-cytoplasmic ratio of of somatotropic cells; V_VGH_ – volume density of somatotropic cells; A_LH_ – area of gonadotropic cells; (N/C)_LH_ – nuclear-cytoplasmic ratio of gonadotropic cells; V_VLH_ – volume density of gonadotropic cells; V_VFS_ – folliculostellate cells in dorsal pituitary. Results are expressed as means ± SD; *p<0.05 vs. young cadavers.

The area of ACTH cells in old cadavers significantly increased, by 12.1%, compared to young cases (Figure 2A) [9]. The volume densities of the ACTH-positive cells statistically significantly increased in old cadavers, by 109.71%, compared to younger cadavers [10] (Figure 2B). These authors [10] showed a statistically significant positive correlation between the age of the examined cases and ACTH-positive cell volume density. Pavlović et al. [10] reported that linear regression analysis showed that the age of studied cases in their sample significantly predicted the volume density of anterior pituitary ACTH cells. Correlation analysis between the age and morphometric parameters of somatotropes showed that there was a significant positive correlation between age and area of somatotropes [28]. Area and volume density of GH cells in old cadavers significantly increased, by 23.22% and 24.68%, respectively (Figure 2C, Figure 2E), and the nuclear-cytoplasmic ratio of GH immunoreactive cells of the anterior pituitary decreased significantly with aging and was lower by 28.35% compared to younger cases (Figure 2D) [14]. Antić et al. [29] showed that the volume density of somatotropes of the anterior pituitary decreases with age. The area of gonado-tropic LH cells in old cadavers significantly increased, by 44.37% (Figure 2F), while the nuclear-cytoplasmic ratio decreased by 31.45% in relation to younger cases (Figure 2G). Volume density of gonadotropic LH changed insignificantly, by 1.3 times, in comparison to younger cases (Figure 2H) [12]. The volume density of FS cells in old cadavers was significantly increased, by 167.01% in the dorsal and by 105.76% in the ventral half of the adenohypophysis, compared to younger cases (Figure 2I) [14]. Linear regression analysis showed that the volume density of FS cells in the examined cases is a statistically significant predictor of the nuclear-cytoplasmic ratio of GH immunoreactive cells [14]. The results of the correlation analysis of the morphometric parameters of LH and FS cells showed that the volume density of FS cells is significantly positively correlated with the surface area of LH cells, while it is negatively correlated with the nuclear-cytoplasmic ratio of LH cells (Figure 2H).

## Conclusions

Changes in the levels of ACTH, gonadotropic LH, somatotropic and folliculostellate cells of the adenohypophysis in men are statistically significantly more pronounced after the age of 70. It can be concluded that during the aging of men, the volume density of ACTH, gonadotropic LH, somatotropic and folliculostellate cells of the adenohypophysis of men increases significantly. There is a statistically significant correlation between some of the analyzed morphometric parameters of gonadotropic LH and somatotropic cells of the adenohypophysis, which indicates the parallel occurrence of adaptation, i.e. compensatory mechanisms in these cells during aging. FS cells can influence the synthesis and release of ACTH, GH, LH cells and HPA activity through various mechanisms. Although the need to uncover hypothalamic-pituitary interactions is critical to understanding health and disease, the unavailability of human hypothalamic and pituitary tissues for research and the complexity of scientific techniques is a major problem for researchers.

## Dodatak

### Acknowledgements

The research described in this paper was financially supported by the Ministry of Science, Technological Development, and Innovation of the Republic of Serbia (Number of grants: 451-03-65/2024-03/200113), for which the authors are grateful.

### Conflict of interest statement

All the authors declare that they have no conflict of interest in this work.
